# Transgenic Zebrafish Expressing Rat Cytochrome P450 2E1 (CYP2E1): Augmentation of Acetaminophen-Induced Toxicity in the Liver and Retina

**DOI:** 10.3390/ijms24044013

**Published:** 2023-02-16

**Authors:** Yoshinori Sato, Wenjing Dong, Tatsuro Nakamura, Naohiro Mizoguchi, Tasuku Nawaji, Miyu Nishikawa, Takenori Onaga, Shinichi Ikushiro, Makoto Kobayashi, Hiroki Teraoka

**Affiliations:** 1 School of Veterinary Medicine, Rakuno Gakuen University, 582, Bunkyodai-Midorimachi, Ebetsu 069-8501, Hokkaido, Japan; 2 Chemicals Evaluation and Research Institute, Japan (CERI), 3-2-7, Miyanojin, Kurume 839-0801, Fukuoka, Japan; 3 Department of Biotechnology, Faculty of Engineering, Toyama Prefectural University, 5180, Kurokawa, Imizu 939-0398, Toyama, Japan; 4 Department of Molecular and Developmental Biology, Institute of Medicine, University of Tsukuba, Tsukuba 305-8575, Ibaraki, Japan

**Keywords:** acetaminophen, bioactivation, CYP2E1, rat, zebrafish

## Abstract

Metabolic activation is the primary cause of chemical toxicity including hepatotoxicity. Cytochrome P450 2E (CYP2E) is involved in this process for many hepatotoxicants, including acetaminophen (APAP), one of the most common analgesics and antipyretics. Although the zebrafish is now used as a model for toxicology and toxicity tests, the CYP2E homologue in zebrafish has not been identified yet. In this study, we prepared transgenic zebrafish embryos/larvae expressing rat CYP2E1 and enhanced green fluorescent protein (EGFP) using a β-actin promoter. Rat CYP2E1 activity was confirmed by the fluorescence of 7-hydroxycoumarin (7-HC), a metabolite of 7-methoxycoumarin that was specific for CYP2 in transgenic larvae with EGFP fluorescence (EGFP [+]) but not in transgenic larvae without EGFP fluorescence (EGFP [−]). APAP (2.5 mM) caused reduction in the size of the retina in EGFP [+] larvae but not in EGFP [−] larvae, while APAP similarly reduced pigmentation in both larvae. APAP at even 1 mM reduced the liver size in EGFP [+] larvae but not in EGFP [−] larvae. APAP-induced reduction of liver size was inhibited by *N*-acetylcysteine. These results suggest that rat CYP2E1 is involved in some APAP-induced toxicological endpoints in the retina and liver but not in melanogenesis of the developing zebrafish.

## 1. Introduction

Species difference is the most important problem in the use of animal models in pharmacological and toxicological testing. Chemicals absorbed by animals are metabolized by phase I enzymes (oxidation, reduction, hydrolysis) and then phase II enzymes (conjugation reaction) for detoxification. Additionally, it has been established that some active metabolites can be formed through metabolic reactions to exert toxicological and pharmacological responses (bioactivation). Cytochrome P450 (CYP), the most important group of metabolic enzymes, is believed to be involved in bioactivation of chemicals for many toxicological responses [1]. Carcinogenicity is best known as chemical toxicity caused through bioactivation by CYP subtypes (CYPs) as some unstable intermediate products of carcinogenic substances by CYPs can attack genomic DNA [2]. Similarly, the liver is a representative target organ of harmful chemicals through bioactivation by CYPs and other enzymes as the liver contains various species of CYP in large amounts [3].

Animal welfare concerns regarding animal experiments become increasingly serious. About ten years ago, major cosmetic manufacturers in the European Union (EU) and other countries declared that they would not use mammalian animals for testing their products [4]. Since the European Citizens’ Initiative demanded an end to animal testing for medical drugs with signatures from more than one million citizens, the European Medicines Agency has implemented new measures to minimize animal testing during the development of medical drugs [5]. In these circumstances, the zebrafish has attracted much attention in EU since experiments with zebrafish are not considered animal experiments until the zebrafish have acquired self-feeding ability, which occurs around 120 h post fertilization (hpf) [6]. Even adult fish are not covered by various guidelines for animal experiments in Japan. Zebrafish embryos/larvae have many additional strong points. Zebrafish embryos can be exposed to small amounts of chemicals and can be observed in plastic dishes under a conventional microscope similar to cultured cells since they are very small, with a body length of less than 1 mm [7]. They develop very rapidly, and primordia of major organs can be completed within 3–4 days post-fertilization (dpf) [8]. Knowledge on ADME (absorption, distribution, metabolism, and excretion) of zebrafish is far from sufficient compared to knowledge on other major experimental animals even though such knowledge is indispensable for extrapolation of pharmacological and toxicological data to the other animals, especially humans [9]. Goldstone et al. [10] established the nomenclature of an enormous number of CYP species in zebrafish for comparison with those in humans and rats. Members of the CYP1A and CYP1B are well conserved in zebrafish, and mammals and zebrafish CYP3C1-4 show homology to human CYP3A4 [10,11]. However, zebrafish CYP2 families are less homologous to those of humans and rats. It was reported by Tsedensodnon et al. [12] that zebrafish CYP2Y3 and CYP2P6 have 43% homology to mammalian CYP2E1. They found immunoreactivity to anti-human CYP2E1 that was induced by ethanol in larval zebrafish. However, they also found that larval zebrafish showed different substrate specificity to mammalian CYP2E1 and that 4-methylpyrozole, an inhibitor specific to CYP2E1, had no effect on CYP2E1-like activity [12].

Acetaminophen (APAP) is one of the most common analgesics and antipyretics. Although APAP is regarded as very safe, it was reported that high dosing of APAP caused hepatotoxicity [13]. It is well known that CYP2E1 is involved in APAP-induced hepatotoxicity through the production of *N*-acetyl-p-benzoquinoneimine (NAPQI) [14]. It is thought that depletion of glutathione (GSH) by NAPQI causes oxidative stress, resulting in hepatotoxicity. APAP toxicity has also been reported in adult zebrafish and larvae [15,16]. North et al. [17] showed that APAP caused reduction of liver size through inducing apoptosis in larval zebrafish and that the combination of prostaglandin E2 (PGE2) and *N*-acetylcysteine (NAC), a precursor of GSH, reversed APAP toxicity in the liver. In their experiments, however, a high concentration of APAP (10 mM) was used. This indicates that a different CYP species or a smaller amount of a CYP2E1 counterpart in zebrafish might be involved in APAP-induced liver toxicity.

Rats have been the most representative animals for toxicological research and testing of chemicals including APAP. The purpose of this study was to generate a transgenic zebrafish line expressing rat CYP2E1 throughout the body in order to detect general effects of chemicals as a more suitable model for pharmacological and toxicological studies including screening tests.

## 2. Results

### 2.1. Generation of Transgenic Zebrafish Expressing Rat CYP2E1

**Figure 1:**
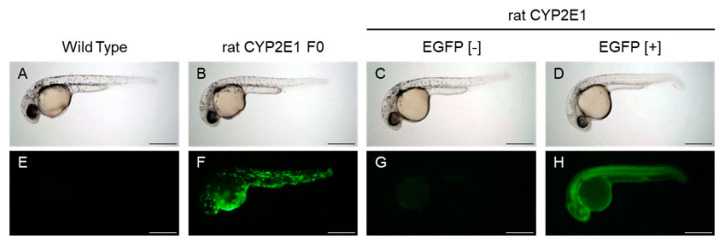
EGFP expression in embryonic zebrafish expressing EGFP and rat CYP2E1. (**A**–**D**) are bright field images of 32 hpf embryos showing EGFP fluorescence in the lower row (**E**–**H**). (**A**,**E**): wild-type embryo. (**B**,**F**): pT2A plasmid-injected embryo (rat CYP2E1 F0). (**C**,**D**,**G**,**H**): transgenic embryo (F2) that was obtained via incross mating between F1 [+/−] adult fish. (**C**,**G**): EGFP-negative embryo (EGFP [−]). (**D**,**H**): Strongly fluorescent embryos (EGFP [+]). Bar indicates 200 µm.

We found EGFP-derived green fluorescence in a mosaic pattern in some embryonic and larval zebrafish that were injected with a pT2A plasmid containing rat CYP2E1 and capped mRNA (cRNA) of transposase at the one cell stage (Figure 1), although the intensity and pattern were variable for each fish. Mosaic expression of EGFP protein in the zebrafish embryo/larva is very common in the case of plasmid injection [18].

EGFP-fluorescence-positive embryos (F0) were kept until the adult stage and outcrossed with WT zebrafish to obtain F1 (Figure S1). Then, F1-male and F1-female adult fish were mated with each other (incross) to obtain embryos/larvae that homogenously express EGFP throughout the body (Figure 1). These embryos/larvae might also express rat CYP2E1 (F2 [+/−] or F2 [+/+]) as well as non-fluorescent (F2 [−/−]) embryos/larvae as negative controls using a fluorescent stereo microscope.

Although β-actin is ubiquitous as a housekeeping gene, EGFP fluorescence seemed to be stronger in the skin than in the retina and liver, as revealed by confocal microscopy (Figure S2).

Rat CYP2E1 transcripts were also detected throughout the body in EGFP-fluorescence positive F2 embryos (referred to as EGFP [+]) but not EGFP-fluorescence negative F2 embryos (referred to as EGFP [−]) via whole-mount in situ hybridization (WISH) (Figure S3A,B). Quantitative RT-PCR (qPCR) confirmed marked rat CYP2E1 expression in EGFP [+] larvae (Figure S3C). Immunoreactivity to anti-rat CYP2E1 was also confirmed in EGFP [+] larvae but not EGFP [−] larvae and wild-type larvae (Figure S3D).

### 2.2. Measurement of CYP2E1 Activity in Transgenic Zebrafish Expressing rat CYP2E1

In order to confirm rat-CYP2E1-dependent metabolic activity in rat CYP2E1 transgenic zebrafish, embryos/larvae were exposed to 1 ppm p-nitrophenol, a specific substrate to CYP2E1, from 24 hpf and harvested at 54 hpf for determination of its O-hydroxy metabolite, p-nitrocatechol (Table 1). As the result, the amount of p-nitrocatechol in EGFP [+] zebrafish was almost ten times higher than that in wild-type zebrafish, while there was no difference in uptake of p-nitrophenol between them (Table 1).

With the aim of comparing EGFP fluorescence intensity and metabolic activity of rat CYP2E1, 7-methoxycoumarin (7-MC)-O-demethylase (MCOD) activity for each individual larva was determined in vivo (Figures S4 and S5). The intensity of blue fluorescence of 7-hydroxycoumarin (7-HC), a fluorogenic metabolite produced by rat CYP2E1 was quantified in EGFP [+] larvae and EGFP [−] larvae at 54 hpf using an inverted fluorescent microscope. Blue fluorescence of 7-HC was subtle but significantly stronger in EGFP [+] larvae than in EGFP [−] larvae (Figure S4). Since EGFP fluorescent intensity varied much more widely, F0 embryos/larvae were also used for the measurement of MCOD activity using rat CYP2E1 and determining the relationship with EGFP fluorescence (Figure S5). The intensity of blue fluorescence derived from 7-HC was significantly higher in 7-MC-exposed EGFP fluorescence-positive F0 larvae (F0) than in wild-type larvae (Figure S5A). While EGFP fluorescence was mosaic-like, 7-HC fluorescence in rat CYP2E1-F0 larvae was rather uniform throughout the body, suggesting that 7-HC is rapidly distributed throughout the body. When 7-HC fluorescence intensity was plotted as a function of EGFP fluorescence intensity for each F0 larva (Figure S5B), a correlation between them was found, suggesting that larvae with stronger EGFP fluorescence have stronger MCOD activity.

Since EGFP fluorescence could reflect MCOD activity by rat CYP2E1, we used only TG-embryos/larvae with very strong EGFP fluorescence as well as those without EGFP fluorescence as negative controls in the following experiments.

### 2.3. Effects of Earlier Exposure to Acetaminophen on Zebrafish Embryos/Larvae

**Figure 2:**
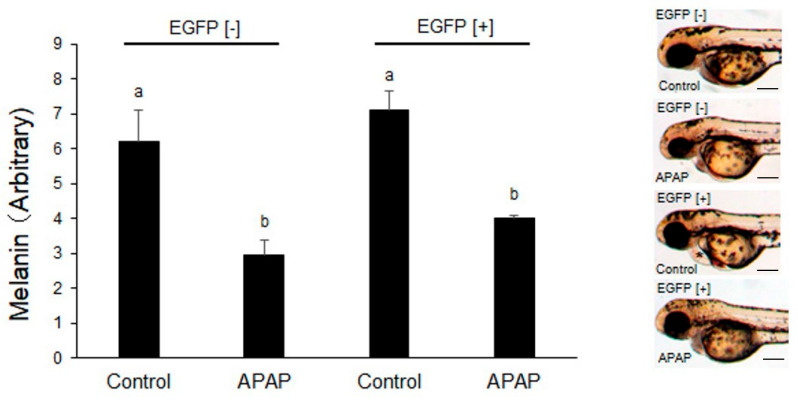
Effects of acetaminophen on melanin contents of rat CYP2E1-TG larvae. EGFP fluorescence-negative (EGFP [−]) and -positive (EGFP [+]) F2 CYP2E1-transgenic larvae were exposed to 2.5 mM acetaminophen (APAP) from 24 hpf to 54 hpf for determination of melanin contents. Control means no treatment since APAP was dissolved in ZR solution. Each tube contained 18–20 larvae, and the melanin contents of three tubes were determined for each treatment (*n* = 3). Bars with different letters (a, b) are significantly different (*p* < 0.05). Inset images in the right are also shown. Asterisk indicates mild pericardial edema in inset image.

When added at 24 hpf, after which rates of natural anomaly and mortality were not found, APAP (2.5 mM) significantly inhibited pigmentation in both EGFP [−] and EGFP [+] embryos/larvae (Figure 2). Melanin contents were quantified at 54 hpf since the same concentration of APAP induced pericardial edema by 54–72 hpf in some larvae irrespective of EGFP fluorescence (asterisk in inset image in Figure 2).

**Figure 3:**
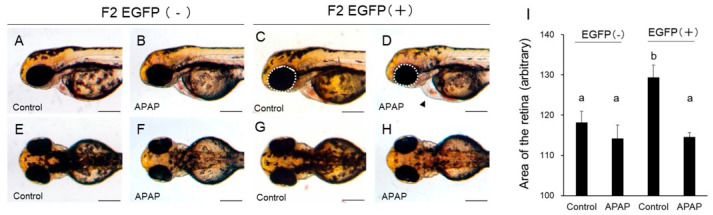
Acetaminophen-induced reduction in the size of the retina in rat CYP2E1-TG larvae. EGFP fluorescence-negative (EGFP [−]) and -positive (EGFP [+]) F2 CYP2E1-transgenic larvae were exposed to 2.5 mM acetaminophen (APAP) from 24 hpf to 54 hpf for capture of lateral and dorsal images. (**A**,**B**,**E**,**F**): EGFP [−] larvae. (**C**,**D**,**G**,**H**): EGFP [+] larvae. (**I**) indicates quantitative data of lateral area of the retina in the bar graph (*n* = 10). The retina was circled by white interrupted line for quantification for example (**C**,**D**). Scale bar: 200 µm. Bars with different letters in (**I**), (a, b) are significantly different (*p* < 0.05). Mild pericardial edema was indicated by arrowhead in D.

Anomalies caused by APAP were not found under a stereomicroscope or an inverted microscope except for reduction in the size of the retina. However, APAP (2.5 mM) induced reduction in the size of the retina at 54 hpf (Figure 3). APAP significantly reduced lateral size of the retina in EGFP [+] larvae compared to the respective controls but not in EGFP [−] larvae. Surprisingly, the size of the retinas of EGFP [+] larvae was significantly larger than in EGFP [−] larvae (Figure 3I). The average of body length of EGFP [+] larvae (1561.1 ± 20.2, *n* = 10) was the same as that of EGFP [−] larvae at 54 hpf (1557.0 ± 10.0, *n* = 10).

### 2.4. Effects of Later Exposure to Acetaminophen on Larval Zebrafish

**Figure 4:**
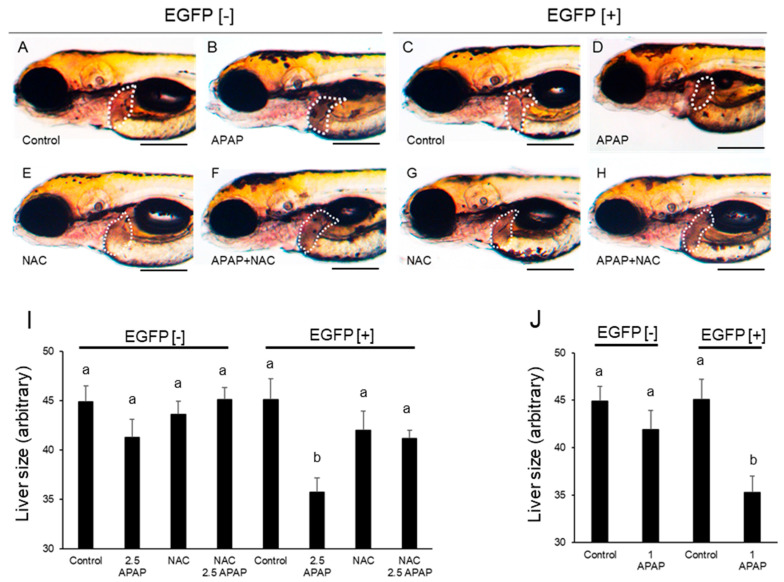
Acetaminophen-induced reduction in the size of the liver in rat CYP2E1-TG larvae and effects of *N*-acetylcysteine. EGFP fluorescence-negative (EGFP [−]) and -positive (EGFP [+]) F2 CYP2E1-transgenic larvae were exposed to acetaminophen (APAP) from 48 hpf to 96 hpf for capture of lateral images (A-H). The white-dotted circle indicates the liver. (**A**,**B**,**E**,**F**): EGFP [−] larvae. (**C**,**D**,**G**,**H**): EGFP [+] larvae. Some larvae were exposed to *N*-acetylcysteine (NAC) (**E**,**G**) and both NAC and APAP at the same time (**F**,**H**). (**I**) indicates averages of areas of the liver in 96 hpf larvae that were exposed to 2.5 mM APAP (2.5 APAP) and/or 20 µM NAC (*n* = 9 or 10). (**J**) indicates averages of areas of the liver in 96 hpf larvae that were exposed to 1 mM APAP (1 APAP) (*n* = 10). Scale bars: 200 µm. Bars with different letters in (**I**,**J**) (a, b) are significantly different (*p* < 0.05).

Following the protocol by North et al. [17], developing zebrafish were exposed to APAP (2.5 mM) at 48–96 hpf. As a result, a significant reduction in liver size in EGFP [+] larvae was found (Figure 4A–I). Reduction of liver size in EGFP [+] larvae was even confirmed in response to 1 mM APAP (Figure 4J). Other toxicological endpoints, including mortality, were not observed after APAP exposure at 96 hpf.

*N*-acetylcysteine is used in treatment of APAP poisoning in humans in order to increase the cysteine in the blood and help in regeneration of glutathione [19]. The effects of NAC on APAP-induced reduction in the size of the liver in EGFP [+] larvae were investigated. As shown in Figure 4A– J, 20 µM NAC effectively prevented reduction in liver size caused by 2.5 mM APAP.

No other anomaly was found in F2 larval zebrafish irrespective of EGFP fluorescence checked under a stereomicroscope and an inverted microscope at 96 hpf.

### 2.5. Effects of APAP on Markers for Hepatocytes and Liver Primordium

**Figure 5:**
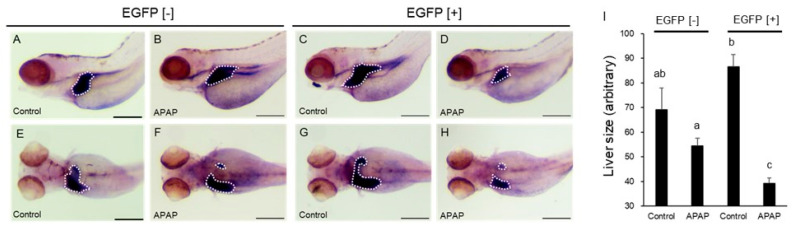
Acetaminophen-induced reduction in the size of the expression area of a hepatocyte marker in rat CYP2E1-TG larvae. EGFP fluorescence-negative (EGFP [−]) and -positive (EGFP [+]) F2 rat CYP2E1-transgenic larvae were exposed to 2.5 mM acetaminophen (APAP) from 48 hpf to 96 hpf. The larvae were fixed for whole-mount in situ hybridization with a fabp10a probe, a hepatocyte marker. The white-dotted circle indicates an outline of the liver. (**A**,**B**,**E**,**F**): EGFP [−] larvae. (**C**,**D**,**G**,**H**): EGFP [+] larvae. (**E**–**H**) are dorsal images of larvae of (**A**–**D**), respectively. Scale bar: 200 µm. (I) indicates averages of the expression area from a dorsal view for each treatment (*n* = 10 or 12). Bars with different letters in I (a, ab, b, c) are significantly different (*p* < 0.05).

The liver type fatty acid binding protein 10a (fabp10a) has been used as a hepatocyte marker for whole-mount in situ hybridization (WISH) [17]. Both EGFP [+] larvae and EGFP [−] larvae that were exposed to 2.5 mM APAP from 48 hpf were used for WISH (Figure 5). Since both the whole body and the liver shrank after fixation with paraformaldehyde, the liver was much smaller than in live larvae and the position was changed (Figure 5A–H). As fabp10a expression was also recognized in the right side in addition to the left side, we quantified the fabp10a expression area using dorsal images. APAP (2.5 mM) significantly reduced fabp10a expression in only EGFP [+] larvae (Figure 5I). The rate of change in fabp10a expression was remarkable compared to the rate of change in liver sizes in live larvae, possibly due to the difference in observations from lateral and dorsal views, while it was difficult to determine the liver in live larvae from the dorsal view.

**Figure 6:**
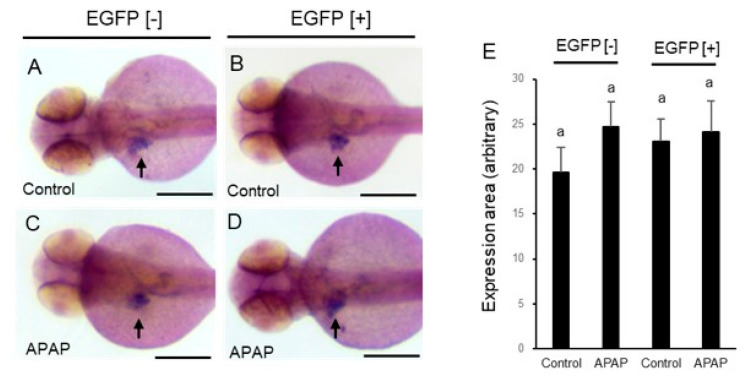
Effect of acetaminophen on hepatocyte differentiation in rat CYP2E1-TG larvae. EGFP fluorescence-negative (EGFP [−]) (**A**,**C**) and -positive (EGFP [+]) (**B**,**D**) F2 CYP2E1-transgenic larvae were exposed to 2.5 mM acetaminophen (APAP) from 48 hpf to 54 hpf. The larvae were fixed at 54 hpf for whole-mount in situ hybridization with a foxa3 probe, a marker for the liver primordium. All images (**A**–**D**) are of a dorsal view, and black arrows indicate the expression area of foxa3. Scale bars: 200 µm. Panel (**E**) indicates averaged areas of foxa3 expression (*n* = 5–9). The “a” over each bar indicates that these values are not statistically different.

We then examined the expression of hepatocyte nuclear factor 3γ (foxa3), a liver primordium marker, at 54 hpf (Figure 6). Expression of foxa3 was confirmed in the liver primordial region in the presence of APAP (2.5 mM), as in the vehicle control (Figure 6A–D). The size of the expression area was not significantly affected by APAP in either EGFP [+] or EGFP [−] larvae (Figure 6E).

## 3. Discussion

Transgenic zebrafish that express rat CYP2E1 in the whole body were established in this study. This model showed rat CYP2E1 activity and can be used to detect various effects of test substances that require bioactivation by CYP2E1 before the development of some specific organs, including the liver. Overall, a group of very fluorescent F2 embryos/larvae (possible F2 [+/+], referred to as EGFP [+]) did not show a notable difference in their early development compared to F2 [−/−] (EGFP [−]) embryos/larvae and wild-type embryos/larvae. They grew to adults normally with sufficient fecundity. Many humanizing zebrafish models have been reported [20], but as far as we know, there has been only one report on transgenic zebrafish that express human or the other mammalian metabolic enzyme [21]. Ingham et al. established humanizing zebrafish that express CYP3A4, one of the most important CYPs in humans, and confirmed metabolic activity accompanying liver development [21]. Since the liver develops in a relatively late stage after the end of formation of primordia of some major organs, it is not always adequate for observation of the effects of active metabolites produced by heterologously expressed human CYP3A on zebrafish development.

Metabolic activity by rat CYP2E1 in EGFP [+] transgenic embryos/larvae was confirmed. Lower but significant hydroxylation of p-nitrophenol, a substrate specific for CYP2E1, and MCOD activity was also detected in wild-type embryos/larvae in this study. Tsedensodnom et al. [12] reported that CYP2E-like immunoreactivity was induced by ethanol, a typical inducer of CYP2E1. Based on nucleotide sequence homology to human CYP2E1 (43%) and inhibition by knock down experiments on APAP-induced steatosis, it is thought that zebrafish CYP2Y3 may be a functional homologue of human CYP2E1 [12]. However, 4-methylpyrazole, a specific CYP2E1 inhibitor, inhibited hydroxylation of 4-nitrophenol, a specific substrate for CYP2E1, at only very high concentrations in ethanol-treated zebrafish larvae [22]. 7-MC is relatively specific for CYP2A6 rather than CYP2E [23]. It was reported that β-naphthoflavone, an aryl hydrocarbon receptor (AhR) agonist, induced MCOD activity in zebrafish larvae at 96 hpf [24]. It is well known that AhR agonists induce CYP3A65 in addition to CYP1A and CYP1B in zebrafish [25]. Although a higher concentration of APAP was able to reduce the liver size in wild-type larvae [17], this may not always have been caused by NAPQI that was produced by CYP2E1-like CYP subtype, since NAPQI was also converted from APAP by human CYP3A4, 1A2, and 2D6 [26]. NAPQI production and conjugates of glutathione and sulfate with NAPQI in larval zebrafish (72–120 hpf) have been reported [15]. Thus, it is unclear whether zebrafish have a CYP subtype that is homologous to mammalian CYP2E1, and it might have different properties in some respects if it exists.

While developmental toxicity induced by APAP is not well known in mammals [27], some researchers have reported toxicological effects of APAP in zebrafish. The number of melanocytes and melanin content was reduced by APAP in larval zebrafish [28]. This was also confirmed in this study, in which APAP exposure started from 24 hpf. APAP inhibited melanogenesis and reduced cell survival rate in cultured human melanocytes [29]. In this study, reduced pigmentation was similarly found in wild-type, EGFP [−] and EGFP [+] embryos, suggesting that pigmentation failure was caused by APAP itself, not by NAPQI. When embryos were exposed to APAP just after fertilization, craniofacial malformation including lower jaw deformity, was also observed in zebrafish [28,30]. Melanocyte distribution is mostly completed by 48 hpf in zebrafish [31]. This malformation is accompanied by an increase of apoptotic cells in the pharyngeal arch. Craniofacial malformation was not found in this study, possibly because our APAP exposure was started in a later stage.

A significant increase in apoptosis in the retina was also reported in APAP-exposed embryos at 48 hpf [30]. We found that the size of the retina was significantly reduced by APAP in EGFP [+] larvae compared to that in vehicle controls but not in EGFP [−] larvae. Although we did not have any data on apoptosis in the retina after APAP exposure in this study, it is well known that many chemicals, including APAP, strongly bind to melanin [29]. Since the retina seems to contain a large amount of melanin, preferential APAP accumulation in the retina could be a possible cause of reduction in the size of the retina. The retina of rats expresses CYP2B1/2 and CYP2C11 but not CYP2E1 [32]. In zebrafish, CYP1B1 is expressed in the embryonic retina [33]. Involvement of CYP1B1 in the production of NAPQI has not been reported as far as we know. While CYP2N13, 2R1, 2Y3, and 3A65 were not detected in the retina of zebrafish at 55 hpf even after exposure to TCDD, rifampicin, and phenobarbital [34], CYP1A was induced by TCDD in the outer layer of the retina of zebrafish [35]. Surprisingly, the size of the retina was larger in EGFP [+] larvae than in EGFP [−] larvae in this study (Figure 3). Although there is no information on the retina, it was reported that the body weight of transgenic mice expressing human CYP2E1 in the liver was consistently larger than that of nontransgenic mice without any explanation for this phenomenon [36]. The body lengths of EGFP [+] embryos/larvae were the same as those of EGFP [−] embryos/larvae of zebrafish even though rat CYP2E1 was expressed throughout the body. Further study is needed to determine the cause of increased retina size by rat CYP2E1 overexpression.

North et al. [17] reported that 10 mM APAP reduced the size of the liver in larval zebrafish. In our experiments, lower concentrations of APAP (1 and 2.5 mM) significantly reduced the liver size in EGFP [+] larvae but not in EGFP [−] larvae at 96 hpf. The rate of reduction in the size of the liver in live larvae seemed to be smaller than the rate of reduction in the size of the fabp10a expression area with WISH due to underestimation of the liver size from the lateral view as the livers of 96 larvae reached the right side of the body. Thus, it was suggested that a metabolite (s) of APAP produced by rat CYP2E1 was involved in the hepatotoxicity in larval zebrafish. Reduction of the liver size might be a result of apoptosis induced by APAP, since it was reported that activity of caspase 3/7 was increased by APAP exposure in wild-type larvae [17]. The expression of foxa3, a marker of the liver primordia [37], was hardly affected by APAP regardless of rat CYP2E1 expression, suggesting that APAP had no effect on hepatocyte differentiation. North et al. [17] reported that the effect of APAP was blocked by NAC as well as PGE2. We also confirmed that NAC inhibited reduction in the size of the liver by APAP in EGFP [+] larvae. NAC is established as an antidote against hepatotoxicity by overdosing APAP via supplementation of glutathione, a major antioxidant substance that is depleted by NAPQI [19]. Involvement of oxidative stress was also supported by a report that APAP-induced hepatotoxicity in zebrafish was inhibited by other antioxidants including a natural medicine (*Forsythiae Fructus*) [38]. Oxidative stress caused by APAP can be derived from Kupffer cells, which were confirmed in the liver of larval zebrafish [39].

## 4. Materials and Methods

### 4.1. Chemicals

Acetaminophen (APAP) (4′-hydroxyacetanilide), *N*-acetyl-L-cysteine (NAC), 7-hydroxycoumarin (7-HC), and 7-methoxycoumarin (7-MC) were obtained from Tokyo Chemical Industry (Tokyo, Japan). 1-Phenyl-2-thiourea (PTU) was purchased from Sigma-Aldrich (St. Louis, MO, USA). All other chemicals were commercially available products of special reagent grade.

### 4.2. Transgenesis of Rat CYP2E1 into the Zebrafish Genome

Transgenesis of rat CYP2E1 into the zebrafish genome was carried out according to Kawakami [40] using a medaka transposon vector (pT2A vector) containing rat-CYP2E1-2A-peptide-enhanced green fluorescent protein (EGFP) linked to medaka β-actin promoter (pT2A/medaka β-actin-p-3xFlag-rat CYP2E1-2A-EGFP). Total RNA was prepared from the liver of a male Wistar rat with TRI reagent (Molecular Research Center, Cincinnati, OH, USA). cDNA was obtained with the resultant total RNA using PrimeScript Reverse Transcriptase (Takara Bio, Otsu, Japan). The first and nested PCR were carried out with Tks Gflex DNA polymerase (Takara Bio) and KOD Dash (TOYOBO, Osaka, Japan), respectively, following the companies’ instructions. The primer sequences used are listed in Table S1. Primers for the nested PCR were attached with restriction enzyme recognition sites for subcloning into the pT2A vector (Figure S6). Nested-PCR products were subcloned into a T-vector (pTAC-2, BioDynamics, Tokyo, Japan) via TA cloning. After complete restriction of the pTAC-2 vector with Hind III and XhoI (New England Biolabs, Ipswich, MA, USA), the resultant open reading frame of rat CYP2E1 was subcloned into the pT2A vector between 3xFlag and 2A peptide using the DNA Ligation Kit (Mighty Mix, Takara Bio) (Figure S6). Capped mRNA (cRNA) of transposase was prepared with the mMESSAGE mMACHINE(tm) SP6 Transcription Kit (Thermo Fisher, Waltham, MA, USA). The original pT2A vector and a transposase plasmid as a template (pCS2 + transpoasese) were kind gifts from Dr. Kawakami.

A mixture of pT2A plasmid (containing rat CYP2E1 and EGFP) and cRNA of transposase was coinjected into the blastomere of one-cell stage embryos (F0) using a fine glass needle connected to an automatic injector (IM-300: Narishige, Japan). Approximately 3 nL of a mixture of 10 ng/µL pT2A plasmid and 10 ng/µL transposase cRNA in Ca^2+^-free Zebrafish Ringer solution (ZR solution: 38.7 mM NaCl, 1.0 mM KCl, 1.7 mM HEPES-NaOH pH 7.2, 2.4 mM CaCl_2_) was injected. EGFP-positive F0 embryos were selected and kept until they became adult fish, which were outcrossed with wild-type fish (WT) to obtain EGFP-expressing F1. F1 adult fish were incrossed to obtain EGFP-positive F2 embryos (TG-fish) for experiments (Figure S1). Bright, fluorescent and confocal microscopic images were obtained with a fluorescent stereomicroscope (MZ-10F, Leica Microsystems, Wetzlar, Germany), an inverted microscope (IX-71, Evident, Tokyo, Japan), and a confocal laser scanning fluorescence microscope (C2- Eclipse Ti, Nikon, Tokyo, Japan).

### 4.3. Measurement of Rat CYP2E1 Activity

Zebrafish embryos (40 embryos/larvae per replication, three replicates per level per time point) were exposed to 1 ppm p-nitrophenol from 24 hpf up to 54 hpf for determination of p-nitrophenol and p-nitrocatechol. The embryos/larvae were washed with ZR solution three times (for about 10 **s** for each wash) in 2.0 mL plastic tubes and stored frozen at −80 °C until further processing. Then, the embryos/larvae were homogenized with a silicone pestle in the 2.0 mL plastic tube containing a mixed solvent of tetrahydrofuran/ultrapure water (1/1 *v*/*v*) to extract the test compound. The mixture was centrifuged at 10,000× *g* for 5 min at room temperature with a refrigerated centrifuge (CR21GII, Hitachi Koki, Tokyo, Japan). The supernatant was collected in a volumetric flask. The extraction procedure was performed twice, and the supernatants from the two batches were mixed and filled up to a volume of 1 mL with the mixed solvent of tetrahydrofuran/ultrapure water (1/1 *v*/*v*) to extract the test compound. They were then filtered through a Millex-LG membrane filter with a 0.2 μm pore size (Merck KGaA, Darmstadt, Germany). The filtrate was diluted with the mixed solvent of tetrahydrofuran/ultrapure water (1/1 *v*/*v*) to prepare an analytical sample. Standard solutions of each test compound with adding mixture of embryos or larvae in the control were used to make each calibration curve. p-Nitrophenol and p-nitrocatechol were measured via liquid chromatography–tandem mass spectrometry (LC-MS/MS) analysis with an LCMS-8060NX triple quadrupole mass spectrometer (Shimadzu, Kyoto, Japan) and a Nexera X2 ultra highperformance liquid chromatograph (Shimadzu) equipped with an L-column2 C8 (length 50 mm, inner diameter 2.1 mm, particle size 5 µm; Chemicals Evaluation and Research Institute, Japan (CERI), Kurume, Japan). Each 10 μL sample was eluted at a flow rate of 0.2 mL/min in the following mobile phase of 5 mmol/L ammonium acetate solution (A, 40%) and 25 mmol/L ammonium acetate in methanol/tetrahydrofuran (1/4 *v*/*v*) (B, 60%). Both chemicals were monitored using an electrospray ionization (ESI) probe. Data were acquired in negative ion mode by using selective reaction monitoring (SRM). The temperatures applied were, for the auto sampler 5 °C and column 40 °C. Three transitions were monitored for p-nitrophenol and p-nitrocatechol: (1) the precursor ion *m*/*z*, (2) product ion *m*/*z*, and (3) collision energy were, respectively, p-nitrophenol and p-nitrocatechol.

7-Methoxycoumarin (7-MC) was used as a fluorogenic substrate that is specific for CYP2 [9,23,24]. Zebrafish embryos/larvae were exposed to 100 µM 7-MC (with 0.01% DMSO) in ZR solution from 48 hpf to 54 hpf in a 3.5 cm plastic petri dish (AGC Technoglass, Yoshida, Japan). After washing twice with ZR solution, lateral images were captured with a fluorescent inverted microscope (IX70, Olympus, Tokyo, Japan) and with a fluorescent stereomicroscope (MZ10 F: Leica, Wetzlar, Germany). Using Image J 1.53t [41], the blue component of each image of a whole zebrafish embryo was extracted and the intensity of 7-HC (fluorescent metabolite) was quantified to calculate MCOD activity. Average value in EGFP [−] larvae without fluorescent dye exposure was subtracted from each value.

### 4.4. Zebrafish and Chemical Treatment

Fertilized eggs were obtained from natural mating of adult zebrafish (long-fin) in our laboratory according to the *Zebrafish Book* [42]. Adult fish and embryos were maintained at 28.5 °C with a lighting schedule of 14 h light and 10 h dark. Eggs were collected within 1 h of spawning, rinsed, and placed into a clean petri dish. At 24 or 48 h after spawning, fertilized embryos were exposed to either APAP and/or 20 µM NAC in 3 mL of ZR solution in 3.5 cm petri dishes (AGC Techno Glass) for the duration of the experiment (10–13 embryos/dish) [35]. APAP and NAC were exchanged with new ones every 24 h until observation (55, 72 and 96 hpf). In some experiments, embryos were exposed to 0.03% 1-Phenyl-2-thiourea (PTU) from 22 hpf to block pigmentation for whole-mount in situ hybridization [42].

### 4.5. Measurement of the Size of the Retina and Liver

For area measurement of the retina, lateral images of a 55 hpf zebrafish embryos/larva were captured with a stereomicroscope connected to a digital camera (WRAYCAM VEX 120, WRAYMER, Osaka, Japan). For area measurement of the liver, lateral and dorsal images of a 96 hpf zebrafish larva were captured similarly to those of the retina. Areas were quantified with Image J 1.53t.

### 4.6. Measurement of Melanin

Melanin contents were determined basically according to Kim et al. [43]. Zebrafish larvae (18–20/tube) were homogenized in 100 μL PRO-PREP Protein Extraction Solution (iNtRON Biotechnology, Seongnam, Korea). After centrifugation at 10,000 rpm for 5 min, 500 µL 1 M NaOH was added and incubated at 98 °C for 10 min. The mixture was then vigorously vortexed, and optical density was measured at 490 nm.

### 4.7. Whole-Mount In Situ Hybridization

Whole-mount in situ hybridization was performed as described previously [34]. The liver-type fatty acid-binding protein 10a (fabp10a) and hepatocyte nuclear factor 3-gamma (foxa3) were amplified by PCR reaction with KOD Dash (TOYOBO) with the primer sets listed in Table S1. This was followed by subcloning to the pTAC-2 TA vector (BioDynamics) and in vitro transcription with SP6 RNA polymerase (New England Biolabs, Ipswich, MA, USA) with the DIG (digoxigenin) RNA Labeling Kit (Sigma-Aldrich) to obtain antisense RNA probes. Four percent paraformaldehyde (PFA)-fixed embryos were hybridized with digoxigenin-incorporated RNA probes at 65 °C overnight. Following hybridization and washing, the embryos were incubated with an anti-DIG antibody conjugated with alkaline phosphatase (Sigma-Aldrich) at 4 °C overnight. Color reaction was performed via incubation in NBT/BCIP Ready-to-Use Tablets substrate (Sigma-Aldrich).

### 4.8. Statistics

Results are presented as means ± SEM. Significant differences between three or more groups were determined using one-way ANOVA followed by the Tukey–Kramer test (*p* < 0.05). Correlation analysis was also carried out for linear relationships between two variables. These analyses were carried out with Excel 2016 with Statcel (the add-in forms on Excel 4th ed.) (OMS Publishing, Tokyo, Japan). Student’s *t*-test was also used to compare means of two groups after the F-test (*p* < 0.05).

## 5. Conclusions

Results of this study confirmed rat-CYP2E1-mediated toxicological endpoints including reduction in sized of the retina and liver in larval zebrafish. Possible developmental toxicity of APAP was confirmed again using the zebrafish model since expression of CYP2E was reported in fetal tissues and the placenta of hamsters and humans [44,45]. Drug-induced liver injury (DILI) is a major reason for post-marketing withdrawals of medical drugs [46]. Now, primary cultured human liver is being used for toxicological testing of chemicals as a gold standard, while there are some problems including a large badge difference. Since it is thought that Kupffer cells (or macrophages), bile ducts, and the vascular system other than hepatocytes are also important targets of DILI, a larval zebrafish that has all of them was proposed as an important model of DILI [39]. Since CYP2E1 is a representative CYP subtype that is involved in bioactivation of some toxicological endpoints, including DILI, our approach is promising for the establishment of a useful screening model in vivo.

## Figures and Tables

**Table 1:** Detection of a rat-CYP2E1-specific substrate and its hydroxyl metabolite in rat CYP2E1-TG larvae.

	p-nitropheonl	p-nitrocatechol
Wild type	1.577 ± 0.191 ^a^	0.052 ± 0.015 ^a^
EGFP [+]	1.560 ± 0.123 ^a^	0.525 ± 0.113 ^b^

Wild-type and rat CYP2E1-TG zebrafish were exposed to p-nitrophenol, a specific substrate for rat CYP2E1, from 24 hpf and harvested at 54 hpf for determination of p-nitrophenol and its hydroxyl metabolite, p-nitrocatechol. Contents of both chemicals were expressed ng/embryo. p-Nitrocatechol content of EGFP [+] larvae were significantly higher than those of wild-type larvae. Different letters (^a^, ^b^) are significantly different to respective control (*p* < 0.05, *t*-test).

## Data Availability

The datasets generated during and/or analyzed during the current study are available from the corresponding author on reasonable request.

## Supplementary Materials

The supporting information can be downloaded at: https://www.mdpi.com/article/10.3390/ijms24044013/s1.
