# Supplementary material for: Transgenic Zebrafish Expressing Rat Cytochrome P450 2E1 (CYP2E1): Augmentation of Acetaminophen-Induced Toxicity in the Liver and Retina
Source: Int J Mol Sci. 2023 Feb 16;24(4):4013. doi: 10.3390/ijms24044013 (PMC9968093; doi:10.3390/ijms24044013)
Supplement: Supplementary file 1 [file ijms-24-04013-s001.zip › ijms-2149038-supplementary.pdf]

Figure S1

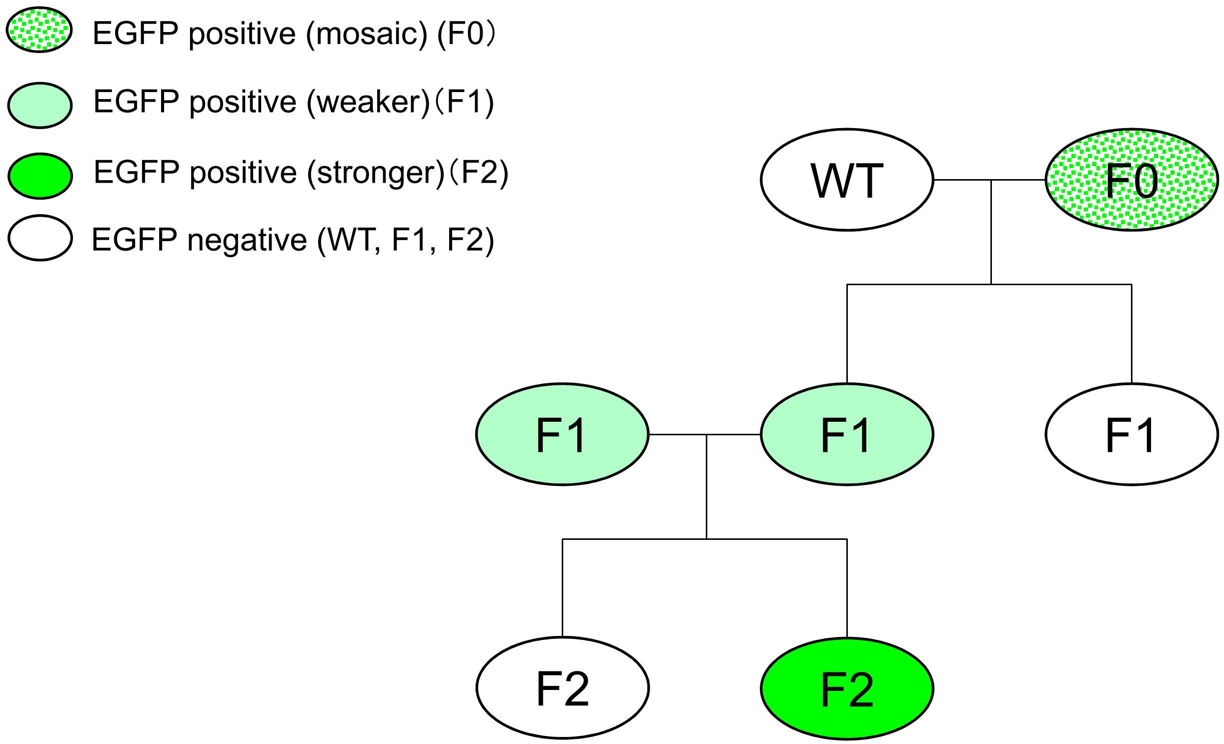

Figure S1. Family trees of EGFP/CYP2E1-expressing zebrafish.

A mixture of pT2A plasmid and transposase cRNA was injected into a blastomere of a one-cell stage embryo by using fine glass needle connected to a microinjector. Rat CYP2E1 and EGFP were incorporated into the zebrafish genome at random. Embryos with EGFP fluorescence in a mosaic pattern (F0) were kept until they became adult fish to be outcrossed with wild-type fish. EGFP-positive embryos (F1, +/−) in their siblings were kept until they became adult fish for incross mating with other EGFP-positive F1 fish to obtain strongly fluorescent F2 embryos for experiments.

Figure S2

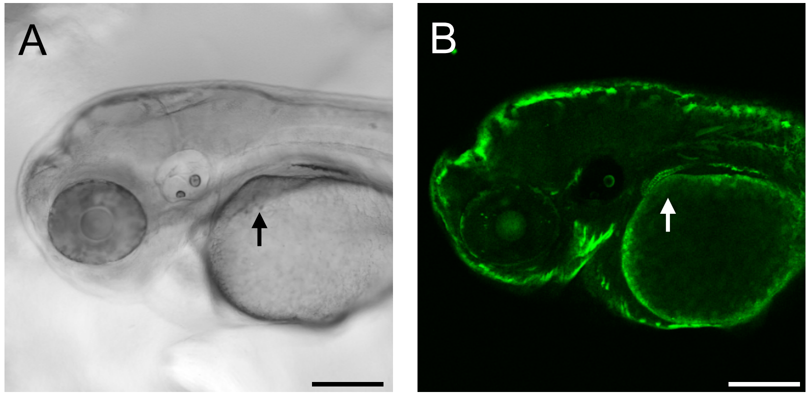

Figure S2. Confocal images of EGFP fluorescence in rat CYP2E1-TG zebrafish in early development.

Bright field image (A) and confocal fluorescent image (B) of the same F2 CYP2E1-transgenic larvae at 72 hpf are shown. Arrows in A and B indicate the liver. Scale bar: 100 µm.

Figure S3

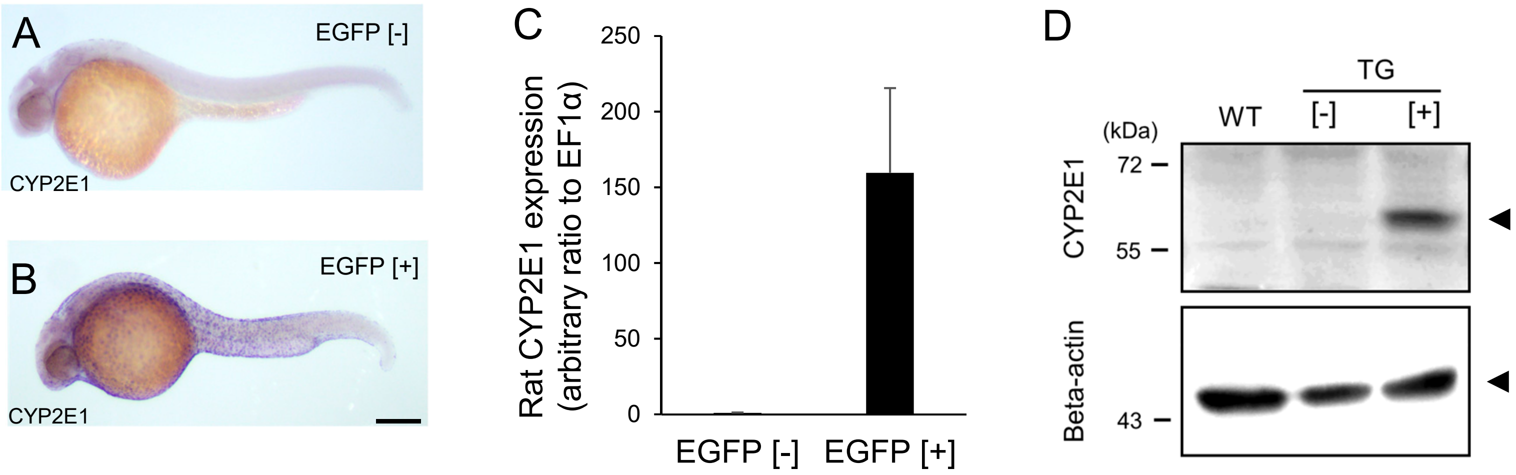

Figure S3. Detection of rat CYP2E1 transcripts and CYP2E1-immunoreactivity in rat CYP2E1 transgenic zebrafish.

A, B: rat CYP2E1-transgenic (TG) zebrafish were fixed at 32 hpf for whole-mount *in situ* hybridization (WISH) with a rat CYP2E1 probe. A: EGFP [−] embryo, B: EGFP [+] embryo. Scale bar: 100 μm. C: Transcripts of rat CYP2E1 in 54 hpf TG fish (EGFP [−] larvae and EGFP [+] larvae) were determined by qPCR. Results are expressed as mean±SEM after normalization with zebrafish elongation factor 1α (EF1α). *n*=3 (25 zebrafish for each case). D: Immunoblot images of homogenate fraction from wild-type (WT), EGFP [−] and EGFP [+] rat CYP2E1-TG zebrafish are shown. Homogenate fraction from 8 zebrafish of 54 hpf was loaded into each lane. Upper and lower panel show immunoblot images to anti-rat CYP2E1 antibody and anti-human β-actin antibody (Abcam), respectively. Arrowheads indicate CYP2E1-immunoreactive band and β-actin-immunoreactive band, respectively. qPCR and immunoblotting were carried out according to Nawaji et al. [34] and Ono et al. [doi: 10.1080/00498254.2018.1483543], respectively. A primer set (rat CYP2E1 situ F TCAGCTGGATTTGAAGGATATCC, rat CYP2E1 situ R TTTTTCCTTCTCCATCTCTATG) were used to prepare RNA probe for WISH. For qPCR of EF1α , a primer set used in the previous paper were used again in this study (doi: 10.1016/j.taap.2008.09.021.). For qPCR of rat CYP2E1 (NM_031543.2), a primer set (rat CYP2E1 qPCR F CAAGTCTTTCACCAAGTTGGCAA, rat CYP2E1 qPCR R TGGCCTTTGGTCTTTTTGAGCT-3′) were used.

Figure S4

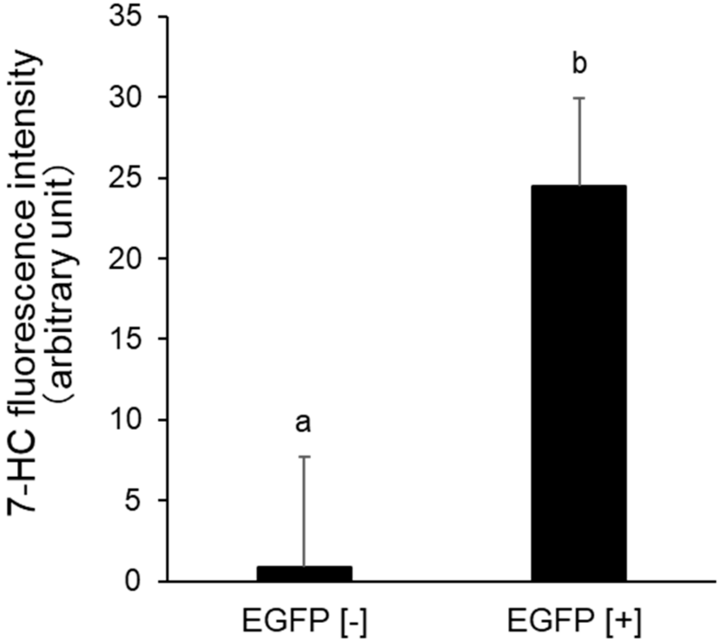

Figure S4. 7-Methoxycoumarin-O-demethylase activity in rat CYP2E1-TG zebrafish.

EGFP fluorescence-negative (EGFP [−]) and -positive (EGFP [+]) F2 CYP2E1-transgenic larvae were exposed to 7-methoxycoumarin (7-MC), a substrate for CYP2E, from 48 hpf and lateral fluorescent images of whole bodies were captured at 54 hpf. The blue component of each image of the whole zebrafish body was extracted and the intensity of 7-hydroxycoumarin (7-HC), a fluorescent metabolite of 7-MC, was obtained to calculate average values for groups of EGFP [−]- and EGFP [+]-larvae (n=13). Bars with different letters (a, b) are significantly different by *t-test* (p< 0.05).

Figure S5

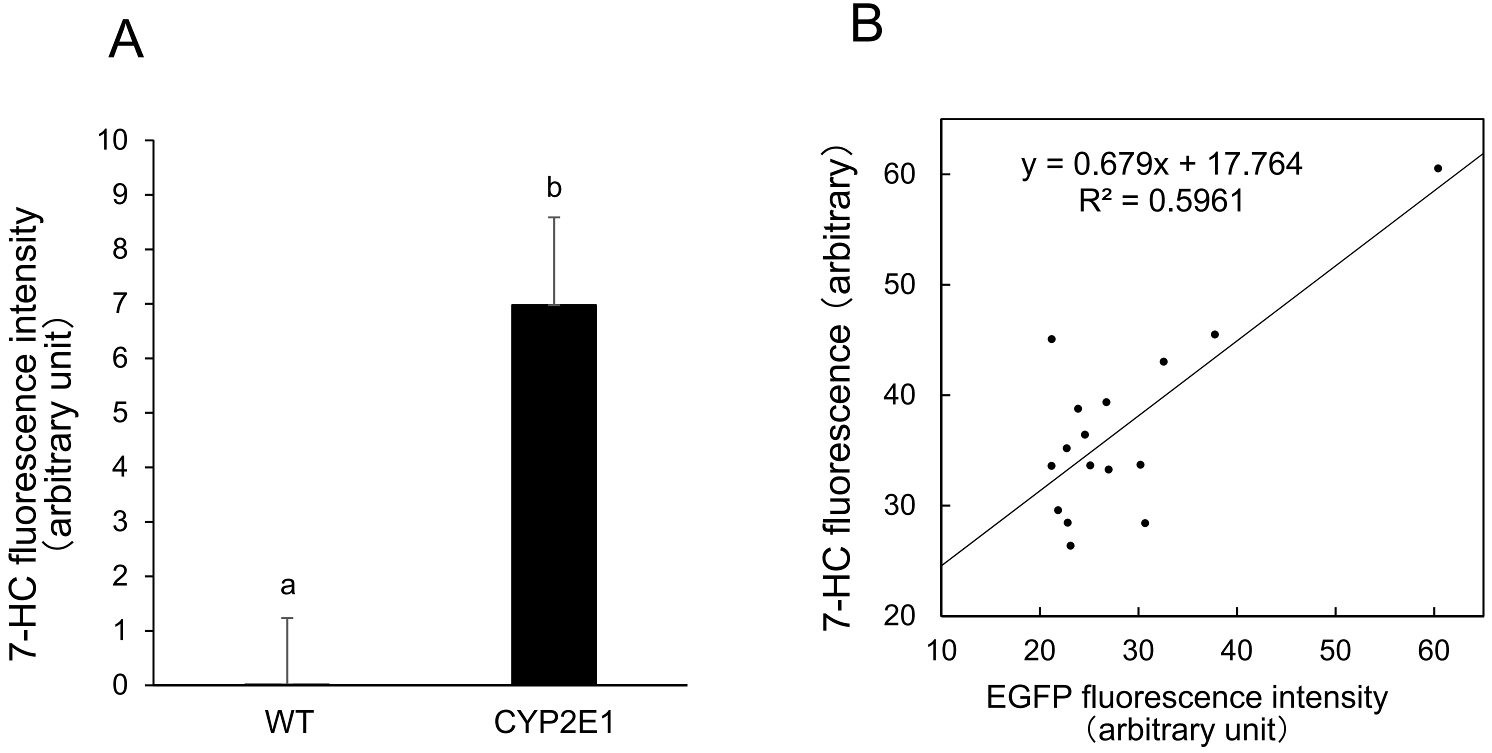

Figure S5. 7-Methoxycoumarin-O-demethylase activity in larval zebrafish expressing rat CYP2E1 (F0).

(A) Larvae of wild-type and pT2A plasmid-injected embryos (F0) were exposed to 7-methoxycoumarin (7-MC), a substrate for CYP2E, from 48 hpf and lateral fluorescent images of the whole bodies were captured at 54 hpf. The blue component of each image of the whole zebrafish body was extracted and the intensity of 7-hydroxylated coumarin (7-HC), a fluorescent metabolite of 7-MC, was obtained to calculate average values for groups of wild-type (WT) and EGFP-fluorescent rat CYP2E1-F0 larvae (CYP2E1) (n=33). Bars with different letters are significantly different by t-test (p<0.05). (B) The green component of each image was extracted as EGFP fluorescence in rat CYP2E1-F0 larvae. A correlation graph between 7-HC fluorescence intensity and EGFP fluorescence intensity was constructed. The correlation line is Y = 0.679X + 17.764 (X is EGFP fluorescence and Y is 7-HC fluorescence.). Correlation coefficient (r) was 0.77.

Table S1

Table S1. Nucleotide sequences of primers used in this study

Primer	Sequence	Purpose
rat CYP2E1-F1	5’-CTGATGAGCCACCCTCCTTC-3’	pT2A vector for transgenesis
rat CYP2E1-R1	5’-ATATCAGGAAGTTTTCAGGTCTC-3’	pT2A vector for transgenesis
rat CYP2E1_Hind III-F2	5’-TATATAAGCTTTAGCGCACATGGCGGTTCTT-3’	pT2A vector for transgenesis
rat CYP2E1-R1_Xho I-R2	5’-TATATCTCGAGATATCAGGAAGTTTTCAGGTCTC-3’	pT2A vector for transgenesis
zebrafish fabp10a-F	5’-ACAGCAGCAGAAGACCACACC-3’	Probe for *in situ* hybridization
zebrafish fabp10a-R	5’-CTCCTCGTAGTTCTCCTGAGC-3’	Probe for *in situ* hybridization
zebrafish foxa3-F	5’-CCTCAACGAAATCTACCAGTG-3’	Probe for *in situ* hybridization
zebrafish foxa3-R	5’-AAACACATTAGGATGCATTGAGG-3’	Probe for *in situ* hybridization

Nucleotide sequences of primers used in this study. Nucleotide sequences of target genes were M20131.1 (rat CYP2E1), NM_152960.1 (zebrafish fabp10a) and NM_131299.1 (zebrafish foxa3) in GenBank.

Figure S6

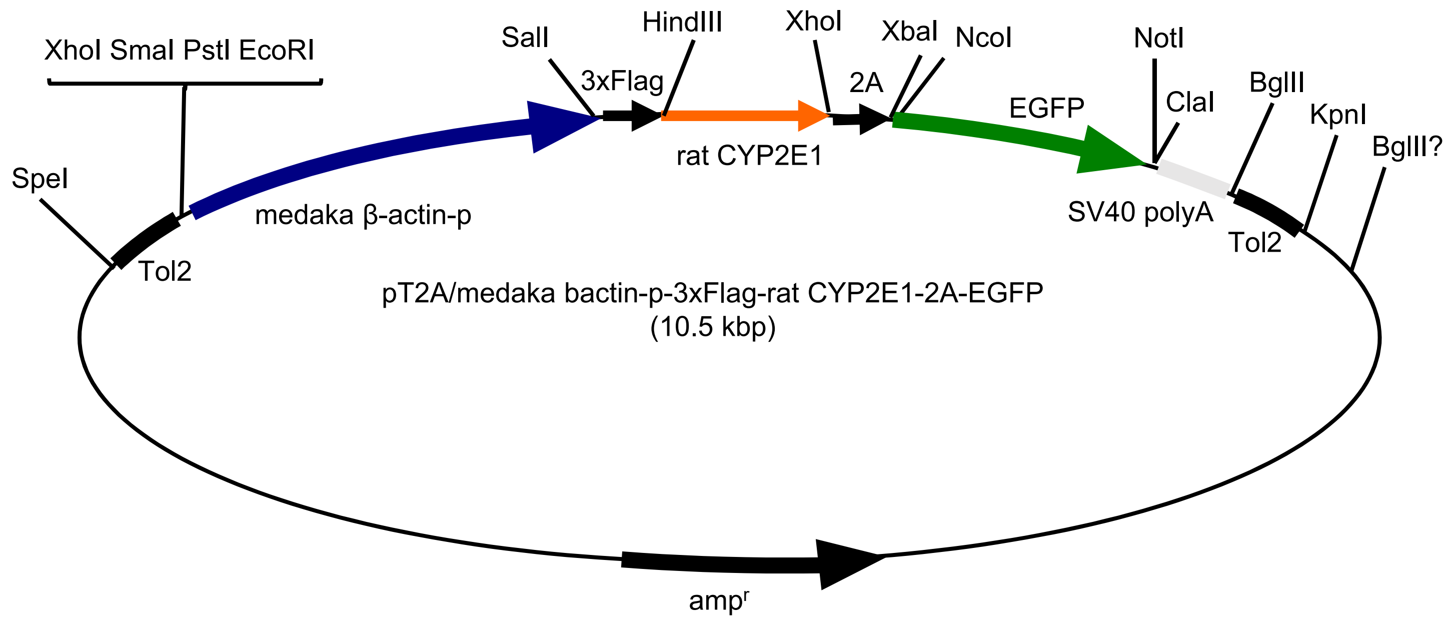

Figure S6. Map of the pT2A plasmid used for transgenesis of rat CYP2E1 into the zebrafish genome.

Tol2 transposon vector (pT2A) containing an open reading frame of rat CYP2E1-2A peptide-EGFP under the control of medaka β-actin promoter (medaka β-actin-p) was prepared.
